# BARRIERS TO SOCIAL ENGAGEMENT OF OLDER IMMIGRANTS IN THE UNITED STATES: A SYSTEMATIC REVIEW OF LITERATURE

**DOI:** 10.1093/geroni/igae098.3155

**Published:** 2024-12-31

**Authors:** Dolapo Adeniji, Vivian Miller, Betty Tonui, Emma Barnhart

**Affiliations:** Indiana State University, Avon, Indiana, United States; Bowling Green State University, Bowling Green, Ohio, United States; Oakland University, Rochester, Michigan, United States; Bowling Green State University, Bowling Green, Ohio, United States

## Abstract

Social engagement is crucial in older adulthood. Social engagement strengthens a sense of belonging, fosters meaningful participation in activities, enhances physical and mental health, alleviates stress and anxiety, and promotes a healthy lifestyle. However, older immigrants often face challenges in engaging socially due to acculturation stress and unfamiliar cultural norms. Using a systematic review of existing literature, this study seeks to identify existing literature that discusses older immigrants’ involvement and participation in community programming, synthesize findings on the accessibility of such programs, and present recommendations for modifications in programs and policies to serve this population best. Guided by the PICO framework and PRISMA guidelines, a systematic review of 11 university databases yielded (N=20) relevant peer-reviewed journal articles after completing a three-stage review process. These articles document social engagement practices identified among various ethnic backgrounds, including older Korean Americans, Chinese Americans, Mexican Americans, and Vietnamese, as well as immigrants and refugees originating from Africa and Asia. Findings suggest that ageism, social disintegration, and language barriers hinder the effectiveness of existing community programs, thus leading to social isolation and loneliness. While our study encompassed various ethnic groups, it is imperative to recognize the potential unique social engagement practices within each of these groups. This acknowledgment is essential in planning community programs to accommodate languages and cultural preferences that can help alleviate social isolation among aging immigrants. Future studies may benefit from a more targeted examination of social engagement practices within distinct ethnic groups to inform targeted interventions.

